# Taming the Inflammation: The Role of Corticosteroids in Pediatric Mycoplasma Pneumonia

**DOI:** 10.3390/children13030333

**Published:** 2026-02-26

**Authors:** Maria Sole Valentino, Costanza Pagliani, Chiara Lovati, Roberta Caiazzo, Crescenzo Coppola, Raffaella Di Tonno, Marta Stracuzzi, Vania Giacomet

**Affiliations:** Pediatric Infectious Disease Unit, Luigi Sacco Hospital, University of Milan, 20157 Milan, Italy; costanza.pagliani@unimi.it (C.P.); chiara.lovati@unimi.it (C.L.); caiazzo.roberta@asst-fbf-sacco.it (R.C.); coppola.crescenzo@asst-fbf-sacco.it (C.C.); ditonno.raffaella@asst-fbf-sacco.it (R.D.T.); marta.stracuzzi@asst-fbf-sacco.it (M.S.)

**Keywords:** corticosteroids, respiratory infections, *Mycoplasma pneumoniae*, pediatric pneumonia

## Abstract

**Highlights:**

**What are the main findings?**

- This narrative review summarizes evidence on corticosteroid use in pediatric *Mycoplasma pneumoniae* pneumonia, including dosage, timing, and clinical outcomes.
- Despite heterogeneous data, corticosteroids—especially in moderate-to-severe or refractory cases—may improve fever resolution, radiological recovery, and inflammatory markers.

**What are the implications of the main findings?**

- A standardized approach to corticosteroid use in *Mycoplasma pneumoniae* pneumonia is lacking; this review highlights the need for well-designed randomized controlled trials.
- Clinicians should balance potential benefits and risks, particularly in young children or when using high-dose or systemic corticosteroids.

**Abstract:**

**Background/Objectives**: To summarize the available evidence on the use of corticosteroids in the treatment of pediatric *Mycoplasma pneumoniae* pneumonia, including severe and refractory forms. **Methods**: We conducted a narrative literature review of studies published between 2000 and 2024 that investigated corticosteroid therapy in children with *Mycoplasma pneumoniae* pneumonia, including various clinical presentations such as severe *Mycoplasma pneumoniae* pneumonia and refractory *Mycoplasma pneumoniae* pneumonia. Both randomized controlled trials and observational studies were included. **Results**: Early administration of corticosteroids, particularly within 24–36 h of hospital admission, was associated with improved clinical outcomes, including faster fever resolution, shorter hospital stay, and enhanced radiological recovery. High-dose regimens (≥5 mg/kg/day) or pulse therapy appeared effective in severe or refractory cases, while inhaled corticosteroids showed benefit in milder forms. Predictive factors for corticosteroid response included elevated C-reactive protein, lactate dehydrogenase, and ferritin levels. The overall safety profile was acceptable, with minimal adverse effects reported in most studies. **Conclusions**: Corticosteroids may play a beneficial role as adjunctive therapy in pediatric *Mycoplasma pneumoniae* pneumonia, especially in selected cases. However, further high-quality studies are required to define optimal timing, dosage, and patient selection.

## 1. Introduction

*Mycoplasma pneumoniae* (*MP*) is a leading cause of community-acquired pneumonia (CAP) in children, transmitted via respiratory droplets. Infection causes direct tissue damage, exacerbates airway hyperreactivity, and contributes to immune dysfunction [1,2]. The incubation period for *Mycoplasma pneumoniae* pneumonia (MPP) typically ranges from 1 to 3 weeks, after which patients often experience a sudden onset of fever, sore throat, malaise, and headache, whereas coryza, commonly seen in influenza, and wheeze are usually absent. Within 3 to 7 days of the initial symptoms, a predominantly nonproductive cough develops, accompanied by fever generally ranging between 38 °C and 39.5 °C [3,4]. Key indicators of increased risk for MPP include age > 5 years, prolonged prodromal symptoms (>6 days), extrapulmonary manifestations (mainly dermatological ones), family with respiratory symptoms, and normal or mildly elevated C-reactive protein (CRP) and procalcitonin (PCT) levels [5,6]. Diagnosis is typically confirmed through serological detection of MP-IgM or molecular testing via polymerase chain reaction (PCR), typically performed on a nasopharyngeal swab [5,7].

Due to its lack of a cell wall, MP is inherently resistant to beta-lactam antibiotics and cannot be visualized using Gram staining [8]; therefore, protein synthesis inhibitors, such as macrolides and tetracyclines, or DNA synthesis inhibitors, such as fluoroquinolones, are usually effective against MP in vitro, and are typically the drugs of choice.

Macrolides are the recommended first-line antimicrobial treatment for MP infections in immunocompetent children [5]; among these, azithromycin is often preferred due to its efficacy and favorable safety profile [9]. Nevertheless, the widespread use of macrolides has contributed to a global increase in macrolide-resistant *Mycoplasma pneumoniae* (MRMP), particularly in East Asian countries such as Korea, Japan, and China, and to a lesser extent in the United States, where resistance rates range from 5% to 15%. This growing resistance has raised concerns regarding the continued effectiveness of macrolide therapy [8,10,11,12,13,14,15]. Consequently, doxycycline has been increasingly adopted as a first-line treatment for MP infections in pediatric populations. Traditionally, tetracyclines have been restricted to children older than 8 years because of concerns regarding dental discoloration and enamel hypoplasia. However, doxycycline differs from older tetracyclines due to its lower affinity for calcium binding. Emerging evidence and updated pediatric infectious disease guidance suggest that short courses (≤21 days) may be considered when clinically indicated, even in younger children, particularly in severe or refractory cases [8]. Recent comparative studies indicate that doxycycline is unlikely to cause clinically significant dental staining or enamel hypoplasia in children under 8 years of age [16,17,18]. Nevertheless, prescribing practices vary across regions, and age-related recommendations remain influenced by local guidelines and risk–benefit considerations.

Fluoroquinolones, such as levofloxacin, are reserved for immunocompromised children, especially those with prior macrolide exposure, due to the higher risk of severe adverse effects [8,19].

However, the overall efficacy of antibiotic therapy in MPP remains controversial, as the disease is often mild and self-limiting, supporting the hypothesis of an immune-mediated pathogenesis. Consequently, a watchful waiting approach may be appropriate in non-severe cases, provided that patients are closely monitored. Antibiotic therapy should generally be reserved for severe presentations, characterized by oxygen saturation below 93%, respiratory distress, feeding difficulties, or signs of dehydration [9]. On the other hand, there have been reports of macrolide-resistant cases that were severe, and sometimes fatal, and did not respond to macrolides or other effective antibiotics [11,12,13,14,15].

Recent studies suggest that adding corticosteroids, administered by inhalation, orally or intravenously, may enhance treatment outcomes in MPP by improving both clinical symptoms and radiographic findings [11,12,13,14,20]. The pathogenesis of MPP is partly driven by an exaggerated immune response, which contributes to lung tissue damage [3]; key players in this response include various cytokines, such as interleukin (IL)-2, IL-8, and IL-18, as well as other immune mediators such as antigen-presenting cells and T cells [21,22,23]. In this context, corticosteroids have been proposed as a potential therapeutic option, especially in moderate and severe cases, to mitigate excessive inflammation and improve clinical outcomes [24]. However, the optimal dose and timing for systemic corticosteroid treatment remain uncertain.

This narrative review evaluates recent findings on corticosteroid therapy in pediatric MPP, with the goal of clarifying clinical indications, optimal dosing strategies, and predictive markers of therapeutic response.

## 2. Materials and Methods

A structured narrative (non-systematic) literature search was conducted in PubMed, Embase, and Google Scholar for English-language articles published between January 2000 and April 2024. Keywords included “*Mycoplasma pneumoniae*”, “children”, “pediatric”, “corticosteroids”, “methylprednisolone”, “glucocorticoids”, and “inhaled steroids”.

Eligible studies comprised randomized clinical trials, observational studies, and meta-analyses investigating corticosteroid therapy in pediatric patients with confirmed or clinically suspected MPP. Case reports, adult-only studies, non-English publications, and studies lacking clear diagnostic criteria or clinical outcome data were excluded.

Titles and abstracts were screened for relevance, followed by full-text evaluation. A total of 28 records were identified through database searching; after removal of duplicates and screening, 21 full-text articles were assessed for eligibility. Seventeen studies met the inclusion criteria and were included in the final qualitative synthesis.

The aim was to summarize and critically appraise the main themes and findings from the existing literature rather than to perform a formal systematic review.

## 3. Results

### 3.1. Patient Groups and Diagnostic Criteria

Patients were classified across studies into various clinical categories of MPP, including typical, refractory, and severe forms, using slightly differing diagnostic criteria.

Seven studies [13,20,25,26,27,28,29] enrolled patients with MPP based on diverse diagnostic approaches. Generally, diagnosis was made through a combination of clinical symptoms—such as fever (≥38.5 °C), cough, respiratory distress, and/or chest pain—and new radiographic findings, including lobar infiltrates or pleural effusion. Laboratory confirmation typically involved serological testing, with either a positive IgM result or seroconversion (a shift from negative or equivocal to positive), as seen in Yang et al. [13], who performed two serological tests during hospitalization.

Additionally, PCR testing was employed on throat swabs (Huang et al. [27]) or nasopharyngeal aspirates (Han et al. [28]). In the study by You et al. [12], cold agglutinin tests were also used as part of the diagnostic process.

Nine studies [12,30,31,32,33,34,35,36,37] focused on patients with refractory *Mycoplasma pneumoniae* pneumonia (RMPP), which was characterized by a lack of clinical improvement despite at least seven days of appropriate antibiotic treatment with macrolides, except for one study, in which failure to respond after three days of macrolide treatment was considered sufficient for RMPP classification. Key indicators of refractory disease included persistent fever (≥38.5 °C), consolidation affecting multiple lung lobes, and progression of pulmonary lesions. Other radiographic signs included increased lung opacity, pleural effusion, necrotizing pneumonia and lung abscesses.

In four studies [38,39,40,41], patients with severe *Mycoplasma pneumoniae* pneumonia (SMPP) were included. These patients were identified based on poor general condition, prolonged fever (for more than 7 days), dyspnea, decreased oxygen saturation (≤92%) in ambient air, tachycardia, rapid breathing and/or lower chest wall indrawing. In some studies, two or more affected lung lobes were identified through chest X-rays, while others considered complications such as pleural effusion and atelectasis. Severe cases often involved significant lung involvement, with more than two-thirds of thoracic segments showing abnormalities. One study also included multi-system involvement and hospitalization exceeding seven days as criteria for SMPP.

The studies included in this review span a broad pediatric age range, from infancy to adolescence, reflecting the heterogeneous clinical impact of MPP across developmental stages.

A detailed synthesis of study methods and results is presented in Table 1.

### 3.2. Clinical and Biochemical Indicators for the Use of Corticosteroids

Several clinical features and laboratory parameters have been investigated as potential indicators for initiating corticosteroid therapy in patients with MPP.

The study by Liu et al. [25] showed that whole pulmonary lobar consolidation and diffuse bronchiolitis-associated lesions on imaging were critical indicators for high-dose corticosteroid therapy; extensive lung consolidation further supported the use of corticosteroids in SMPP, according to the study by Han et al. [28]. In the study by Zhu et al. [32], lung consolidation and pleural effusion were identified as predictors of methylprednisolone pulse therapy (≥200 mg/day) in RMPP.

Persistent high fever lasting more than five days was another significant factor in determining corticosteroid intervention [25], also in a study including patients with SMPP [28].

Additionally, certain biomarkers have been identified as possible predictors for the use of corticosteroid therapy in the management of MPP.

CRP levels greater than 30 mg/L, and especially exceeding 100 mg/L, were often indicative of corticosteroid initiation in SMPP, including methylprednisolone doses ranging from 2 mg/kg/day to pulse therapy of 10–15–30 mg/kg/day [25]. According to Zhu et al., a CRP level of ≥44.45 mg/L was a key predictor for methylprednisolone pulse therapy of ≥200 mg/day [32].

With regard to lactate dehydrogenase (LDH), levels exceeding 478 IU/L in SMPP and ≥590 IU/L in RMPP have been associated with the use of corticosteroid therapy [28,32]. Finally, ferritin levels of ≥411 ng/L and a neutrophil percentage of ≥73.75% were additional biomarkers predicting methylprednisolone pulse therapy (≥200 mg/day) in RMPP [32].

### 3.3. Scheme and Type of Molecule

#### 3.3.1. Intravenous Methylprednisolone

The most frequently used corticosteroid across the various studies is intravenous methylprednisolone. However, both the dosages and the patient populations varied among the studies (Table 2).

In many cases, the dose used was 1–2 mg/kg/day, administered for 3 to 7 days, in patients with MPP, RMPP, and SMPP [13,25,27,28,30,31,32,33,34,36,38,40,41]. Studies by Yang et al. [13] and Huang et al. [27] adopted an early corticosteroid approach, administering treatment within 24–36 h of admission. This strategy led to significant clinical benefits. In the study by Yang et al. [13], involving 257 children, 74% achieved defervescence within 24 h and 96% within 72 h of initiating corticosteroid therapy. No patients progressed to RMPP, and none required ICU admission. Additionally, an “add-on” strategy was applied for patients not responding within 36–48 h, with high-dose intravenous methylprednisolone (5–10 mg/kg/day), followed by tapering to oral prednisolone after defervescence.

Similarly, Huang et al. [27] conducted a randomized trial in 106 children with SMPP, comparing corticosteroid initiation within 24 h of admission (early group) versus after 72 h (control group). The early-treatment group had a significantly shorter fever duration, shorter hospital stays, and faster radiographic resolution, with only 1.9% showing persistent abnormalities beyond 4 weeks versus 17.5% in the control group.

Therapy duration varied among studies. For instance, Wu et al. [40] administered methylprednisolone for 3 to 5 days, while Zhu et al. [32] extended treatment up to 7 days. In both Yang et al. [13] and Huang et al. [27], corticosteroid doses were tailored to clinical response, with gradual tapering to prevent inflammatory rebound.

In some studies, higher dosages, up to 10 mg/kg/day, were adopted for patients diagnosed with SMPP or RMPP [13,25,27,39,41]. One notable example is the study by Okumura et al., which compared high-dose corticosteroid therapy (≥2 mg/kg/day, mean: 3.5 ± 1.1 mg/kg/day) with low-dose therapy (<2 mg/kg/day, mean: 1.2 ± 0.3 mg/kg/day). The results showed that high-dose treatment in children with RMPP led to a faster resolution of fever, shorter hospital stays, and an overall reduced duration of corticosteroid use [33].

Pulse therapy was investigated in some studies, in cases of SMPP or RMPP. This approach involved high doses of methylprednisolone, up to 30 mg/kg/day for 3 days [12,25,28,37,41] except for the study by Zhu et al. that considered pulse therapy a dose ≥ 200 mg/day regardless of body weight [32]. Pulse therapy is aimed at controlling the excessive immune response seen in severe cases and has been associated with rapid and substantial clinical improvement. What stands out from the study by Liu et al. [25] is that early administration of high-dose corticosteroids (2 mg/kg/day of intravenous methylprednisolone, initiated within 24–36 h of admission) was particularly effective in severe cases marked by persistent fever lasting more than 7 days, CRP levels exceeding 100 mg/L, or lobar consolidation on imaging. These patients showed significantly shorter durations of fever, hospitalization, and radiographic recovery compared to those treated later.

#### 3.3.2. Other Corticosteroid Agents

In addition to methylprednisolone, as described in the meta-analyses by Qiu and Kim, other intravenous corticosteroids have also been used. These include dexamethasone, administered at dosages of 0.2–0.5 mg/kg/day for 4–5 days or 0.2–0.3 mg/kg/day for 5 days, particularly in children with RMPP [36,37], and hydrocortisone, as reported in the study by Okumura et al. [33].

Oral prednisolone was another corticosteroid used, typically at a dose of 1–2 mg/kg/day for 5–7 days, although in some cases the treatment duration was shorter. While this regimen was primarily adopted for mild cases, as reported in the studies by Yang et al. [13] and Han et al. [28], it was also employed in the management of RMPP. For instance, Luo et al. [35] demonstrated that oral prednisolone at 2 mg/kg/day (divided twice daily) combined with intravenous azithromycin for 5 days was significantly more effective than azithromycin alone in the treatment of children with RMPP. All patients in the corticosteroid group achieved defervescence within 48 h, compared to none in the control group. Additionally, the combination therapy led to a shorter duration of hypoxemia and faster resolution of dyspnea. Radiological improvements were also more frequent, including higher rates of infiltration absorption (80% vs. 21.4%), atelectasis resolution (71.4% vs. 12.5%), and pleural effusion disappearance (88.9% vs. 20%), with all differences reaching statistical significance. Finally, among the studies reviewed, two investigations specifically highlight the clinical benefit of combining inhaled budesonide with antibiotic therapy in the treatment of MPP in pediatric populations. Zhang et al. [20] demonstrated that azithromycin sequential therapy—initiated intravenously and followed by oral administration—combined with nebulized budesonide (1 mg BID for 2 weeks) resulted in a significantly higher overall efficacy rate compared to azithromycin alone. The combination group experienced faster resolution of fever, cough, abnormal heart rate, and lung rales, as well as greater improvements in pulmonary function parameters (PEF, FVC, FEV1, PEF25) and inflammatory markers (PCT, IL-6, G-CSF, TNF-α). Immunoglobulin profiles also improved, with increased IgG and reduced IgA/IgM levels, and chest CT imaging showed more marked recovery. Complementing these findings, Zhao et al. [29] reported that the addition of inhaled budesonide to azithromycin, administered via various routes, significantly enhanced overall treatment efficacy compared to azithromycin alone. The combination therapy led to a faster resolution of clinical symptoms: fever (−2.0 to −3.2 days), cough (−2.0 to −3.8 days), and pulmonary rales (−2.0 to −3.0 days). Inflammatory markers such as IL-6, CRP, and TNF-α were significantly reduced, while pulmonary function indices including FEV1, FVC, and PEF improved substantially. Importantly, this improved efficacy was not associated with a higher incidence of adverse events.

### 3.4. Safety and Adverse Effects of Corticosteroid Therapy in Mycoplasma pneumoniae Pneumonia

Several studies have explored the safety profile of corticosteroid therapy in MPP, with varying conclusions.

In a meta-analysis by Zhao et al. [29], the combination of inhaled budesonide and azithromycin did not increase the incidence of adverse events compared to azithromycin alone. Similarly, Zhao Q. et al. [26] reported no statistically significant difference in adverse event rates between the corticosteroid group (3.33%) and the control group (6.67%). Shan et al. [34] observed no adverse reactions in patients treated with methylprednisolone or IVIG.

In contrast, Zhu et al. [32] noted potential risks during methylprednisolone pulse therapy (≥200 mg/day), including 1 case of circulatory instability and 1 case of gastrointestinal bleeding in the high-dose group. However, no significant difference in overall complication rates was found between the pulse and low-dose groups.

Although Zhou et al. [30] raised concerns about the theoretical risk of adverse effects in younger children, no specific adverse events or frequencies were reported. As such, while caution is advised in this age group, the findings do not indicate any clinically evident safety signals associated with short-term corticosteroid use in the studied cohort.

## 4. Discussion

The management of pediatric MPP requires balancing effective antimicrobial therapy with appropriate modulation of the immune response. Corticosteroids have been increasingly explored as a potential adjunctive treatment in pediatric MPP, particularly in cases that are refractory or severe. Available studies suggest that early corticosteroid administration—ideally within 24 to 36 h of hospitalization—may be associated with improved clinical outcomes, including faster defervescence, reduced hospital stays, and better radiologic resolution. Laboratory and radiographic markers such as elevated CRP, LDH, ferritin, and neutrophil count may serve as potential predictors of treatment response. The integration of these biomarkers, inflammatory mediators, clinical indicators, and bedside imaging tools such as lung ultrasound may support risk stratification and closer monitoring of disease evolution, particularly in severe or refractory cases. However, no validated biomarker thresholds currently exist to guide treatment escalation.

Although dosing regimens are widely reported, clear criteria for escalation from low-dose to high-dose or pulse therapy remain undefined. In practice, escalation is generally considered in children with persistent fever beyond 48–72 h of appropriate antimicrobial therapy, progressive radiologic findings, rising inflammatory markers, or clinical deterioration. Rather than implying treatment standardization, a pragmatic clinical approach may guide decision-making. In non-severe hospitalized MPP with persistent symptoms or significant inflammatory response, low-dose corticosteroids (1–2 mg/kg/day) may be considered as adjunctive therapy. In more severe disease characterized by respiratory compromise or marked systemic inflammation, higher doses (≥5 mg/kg/day) may be used. In RMPP—defined by persistent fever and radiologic progression despite adequate antimicrobial therapy—short courses of pulse methylprednisolone (e.g., 10–30 mg/kg/day) have been described. These approaches, however, are largely derived from observational data and regional practice patterns and should be individualized based on clinical judgment; future studies are needed to determine whether biomarker thresholds can reliably guide escalation and to validate structured treatment algorithms.

Beyond pulmonary involvement, *Mycoplasma pneumoniae* infection may also present with extra-pulmonary manifestations, including dermatologic complications such as *Mycoplasma pneumoniae*-induced rash and mucositis (MIRM), neurologic, and hematologic involvement. These manifestations, reported in up to 25–60% of cases, are thought to be primarily immune-mediated rather than due to direct pathogen invasion [42]. Given the immunomodulatory properties of corticosteroids, their use in severe extra-pulmonary presentations is biologically plausible and frequently described in clinical practice. However, current evidence is limited to case reports, small series, and retrospective analyses [43,44]. There are no randomized controlled trials specifically evaluating whether corticosteroids reduce the incidence, severity, or duration of extra-pulmonary complications, including reactive infectious mucocutaneous eruption (RIME)/MIRM. Therefore, while corticosteroids may be considered in selected severe cases, particularly those with significant mucocutaneous involvement, their true impact on these outcomes remains uncertain and warrants further investigation [42,43].

Among available corticosteroids, intravenous methylprednisolone is the most frequently studied agent, with dosing strategies often adjusted to clinical severity. While conventional doses of 1–2 mg/kg/day are commonly used in mild to moderate cases, some studies suggest that high-dose (≥5 mg/kg/day) or pulse therapy (up to 30 mg/kg/day for 3 days) may offer additional clinical benefits in more severe presentations. For example, Okumura et al. [33] and Sun et al. [41] reported that high-dose therapy could lead to improved resolution of fever, cough, and radiologic abnormalities, without a proportional increase in adverse events; however, these findings stem from limited-sample studies and should be interpreted with caution. Inhaled corticosteroids, particularly budesonide, may also be beneficial in milder cases, offering localized anti-inflammatory effects with minimal systemic exposure. Preliminary data support the potential role of inhaled corticosteroids when combined with macrolide-based regimens to enhance symptom control and inflammatory resolution, although further validation is needed.

Despite encouraging findings, the current body of literature is limited by small sample sizes, variability in study design, heterogeneity in treatment regimens, and inconsistent outcome reporting. Many studies included concomitant therapies such as macrolides, intravenous immunoglobulin, or inhaled corticosteroids, which may confound the interpretation of corticosteroid-specific effects. Moreover, most studies rely on surrogate or intermediate endpoints—such as time to defervescence or radiologic resolution—which may not fully capture meaningful long-term clinical improvement.

Overall, the quality of evidence remains moderate to low, given the predominance of retrospective designs, the regional concentration of studies (mainly from East Asia), and the potential for publication bias. An additional source of heterogeneity lies in the diagnostic criteria used across studies. The diagnosis of MPP was variably based on serology, PCR testing, clinical and radiologic findings, or combinations of these approaches. Differences in diagnostic certainty, timing of testing, and definitions of refractory or severe disease may have influenced patient selection and treatment response. Consequently, direct comparability across studies is limited, and the true magnitude of corticosteroid effectiveness may be either overestimated or underestimated.

The overall safety profile of corticosteroids in pediatric MPP appears acceptable, particularly for inhaled formulations. However, potential risks associated with systemic and high-dose corticosteroid therapy should be carefully considered. Known adverse effects include hyperglycemia, hypertension, gastrointestinal bleeding, behavioral changes, and increased susceptibility to secondary infections. Importantly, most available studies are limited by small sample sizes and short follow-up periods, reducing their power to detect uncommon or delayed adverse events. Furthermore, long-term safety data in pediatric populations—particularly regarding growth, bone metabolism, and immune modulation—remain scarce. These considerations underscore the need for cautious patient selection and close monitoring when high-dose or pulse regimens are employed.

Well-designed multicenter randomized trials are needed to better define optimal dosing, timing, duration, and long-term safety. A precision medicine approach—guided by early diagnostics and biomarker stratification—could enhance treatment targeting, reduce risks, and improve outcomes for children with MPP.

## 5. Conclusions

Corticosteroids may provide therapeutic benefit in selected pediatric cases of MPP, particularly when disease is severe or refractory. However, current evidence is heterogeneous, largely observational, and often based on surrogate endpoints such as fever resolution. Clear criteria for treatment escalation and validated biomarker thresholds are lacking, and long-term safety data, especially for high-dose regimens, remain limited. Well-designed multicenter randomized trials are needed to define optimal indications, dosing strategies, and clinically meaningful outcomes.

## Figures and Tables

**Table 1:** Use of corticosteroids in pediatric *Mycoplasma pneumoniae* pneumonia: main findings.

References	Study Region (Country)	Study Design and Methods	Population	Main Findings
Huang L. et al. [27] 2014	China	Randomized clinical trial	106 patients (average age of study group = 5.7 ± 2.5 and of control group 6.1 ± 2.3) with MPP	Patients receiving early corticosteroid therapy (methylprednisolone 1 mg/kg every 12 h within 24 h of admission, followed by a 1-week prednisolone taper), in combination with azithromycin (10 mg/kg/day) and a third-generation cephalosporin (80 mg/kg/day), had significantly shorter hospital stays (*p* = 0.001), total fever duration, and post-steroid fever duration (*p* < 0.01) compared to controls who received corticosteroids within 72 h. Radiographic resolution was faster in the early-treatment group, with fewer patients showing persistence beyond 4 weeks (1.9% vs. 17.5%; *p* = 0.038). On day 4, CRP declined and lymphocyte percentages increased more sharply in cases. On day 7, CD3+ CD4+ T-cell levels were significantly lower in cases vs. controls (31.5 ± 7.4 vs. 35.7 ± 8.9; *p* = 0.01).
Shan LS et al. [34] 2017	China	Open, randomized, controlled clinical trial	168 patients aged 2–13 years with RMPP	The average duration of fever after treatment was significantly shorter in Group A (azithromycin + IV methylprednisolone 2 mg/kg/day for 3 days) and Group B (azithromycin + IVIG 400 mg/kg/day for 3 days) compared to Group C (azithromycin alone 10 mg/kg/day IV for 3 days) (*p* < 0.001), with Group A showing the most rapid fever resolution. Both Groups A and B also demonstrated significantly higher rates of pulmonary infiltration absorption, atelectasis resolution, and pleural effusion disappearance compared to the control (*p* ≤ 0.007). Additionally, CRP, D-dimer, and LDH levels were significantly lower in the combination therapy groups than in Group C (*p* < 0.001).
Luo Z et al. [35] 2014	China	Randomized Controlled Trial	58 patients (average age of treatment group = 7.9 ± 4.1 and of control group 7.6 ± 4.5) with RMPP	All patients in the treatment group (oral prednisolone 2 mg/kg/day in two divided doses plus intravenous azithromycin 10 mg/kg/day for 5 days) achieved defervescence within 8–48 h, compared to none in the azithromycin-only group. The duration of hypoxemia was significantly shorter in the treatment group (1.9 ± 0.9 vs. 2.7 ± 1.1 days, *p* < 0.05), as was the time to dyspnea resolution (1.5 ± 0.7 vs. 2.9 ± 0.6 days, *p* < 0.05). Radiologic improvements at day 7 were markedly better in the treatment group: infiltration absorption (80% vs. 21.4%), atelectasis resolution (71.4% vs. 12.5%), and pleural effusion disappearance (88.9% vs. 20%) (*p* < 0.05 for all). Serum ferritin and LDH levels also decreased significantly. No adverse events or post-treatment complications were observed.
Zhao Q et al. [26] 2022	China	Semi-randomized controlled trial	86 patients aged 2–9 years with MPP	Combination therapy (azithromycin 10 mg/kg/die + methylprednisolone sodium succinate, first dose 2 mg/kg/die and after 3–5 days reduced to 1 mg/kg/die, for 3 days) led to significantly better clinical outcomes (*p* < 0.05), including faster symptom resolution and improved lung function (FVC, FEV1, FEV1/FVC) compared to azithromycin alone (10 mg/kg/die for 5 days, then 4-day rest, then the same treatment). It more effectively reduced pathogen persistence and inflammatory markers (IL-6, TNF-α, CRP, CD8+), and increased CD4+ and CD3+ levels. No significant difference in adverse event incidence (*p* > 0.05).
Zhou H et al. [30] 2022	China	Randomized Controlled Trial	102 patients aged 5–13 years with RMPP	Combination therapy of Azithromycin + methylprednisolone (IV: 2 mg/kg/day for 5 days, then 1 mg/kg/day for 2 days) significantly shortened cough resolution time (*p* = 0.033), lung shadow disappearance (*p* = 0.008), fever duration (*p* = 0.004), and hospital stay (*p* < 0.001) compared with azithromycin alone. It also reduced FeNO and eosinophil levels more effectively (*p* < 0.001). No significant difference in adverse events between groups (*p* = 0.373).
Yang EA et al. [13] 2019	South Korea	Prospective study	257 patients aged 5 months–15 years with MPP	Early corticosteroid therapy (oral prednisolone 1 mg/kg/day or IV methylprednisolone 1–2 mg/kg/day for mild cases; 5–10 mg/kg/day IV for severe cases) resulted in rapid defervescence in 74% of patients within 24 h and 96% within 72 h of treatment initiation. An additional pulse dose of high-dose methylprednisolone was administered if fever persisted beyond 36–48 h or in cases of clinical deterioration. No patients progressed to RMPP, and none required intensive care admission. Fever duration did not differ significantly between patients treated with β-lactams alone and those who also received clarithromycin (5.6 ± 2.8 vs. 5.5 ± 2.9 days; *p* = 0.621).
Zhang H et al. [20] 2023	China	Case–control study	108 patients (average age of study group = 7.76 ± 1.41 years and of control group 7.68 ± 1.23 years) with MPP	Azithromycin + budesonide inhalation (1 mg BID) showed a higher effectiveness rate (96.3% vs. 81.5%, *p* < 0.05) and faster symptom resolution (fever, cough, rales, heart rate). Greater post-treatment improvement in immune, pulmonary, inflammatory, blood gas parameters, and chest CT findings compared to azithromycin alone (*p* < 0.05).
Liu J et al. [25] 2023	China	Observational study	210 patients aged 1 year 3 months–16 years 1 month with MPP	In Group A (bronchiolitis-associated lesions or ground-glass opacities), 8 patients with bilateral diffuse bronchiolitis received very high doses of methylprednisolone (5–15 mg/kg/day); 13/15 showed mild–moderate obstructive dysfunction during recovery. After 3 months, HRCT was normal in 56/59. In Group B (pulmonary segmental/lobar consolidation), 20 patients with lobar consolidation received high-dose methylprednisolone therapy (5–30 mg/kg/day); 19/22 had obstructive dysfunction during recovery. After 3 months, 7 patients showed incomplete radiographic resolution.
Han HY et al. [28] 2021	South Korea	Observational clinical study	56 patients aged 1–15 years with MPP	All patients received early corticosteroid treatment within 24–36 h of admission. 46 patients received low-dose corticosteroids (oral prednisolone 1 mg/kg/day or IV methylprednisolone 1–2 mg/kg/day), while 10 patients with more severe symptoms received high-dose therapy (5–10 mg/kg/day). Following treatment, defervescence occurred in 75% of patients within 24 h, 94.6% within 48 h, and 96.4% within 72 h. No significant differences in fever resolution were observed between macrolide-resistant and macrolide-sensitive groups.
You SY et al. [12] 2013	South Korea	Retrospective study	12 patients aged 3–13 years with RMPP	Rapid improvement in clinical and radiological findings was observed following intravenous methylprednisolone pulse therapy at 30 mg/kg once daily for 3 days. All patients achieved defervescence within 2 h (*p* < 0.001), radiological resolution occurred within an average of 2.6 ± 1.3 days, and CRP levels significantly decreased within 3.0 ± 1.1 days after corticosteroid initiation (*p* < 0.001).
Fang C et al. [39] 2022	China	Retrospective study	120 patients aged ≤12 years with SMPP	Combination therapy (antibiotics with methylprednisolone sodium succinate 10 mg/kg/day intravenous drip for 5 days) significantly reduced time to fever resolution, cough relief, rales disappearance, and hospital stay compared with azithromycin alone (*p* < 0.05). ESR, CRP, IL-6, and CD8+ levels were lower, while CD4+ and CD4+/CD8+ ratios were higher post-treatment (*p* < 0.05). D-dimer levels negatively correlated with pediatric critical illness scores (*p* < 0.05).
Zhu R et al. [32] 2022	China	Retrospective cohort study	59 patients aged 1–36 months with SMPP	Children treated with pulse-dose methylprednisolone (≥200 mg/day) showed more severe clinical symptoms, including hypoxemia, extrapulmonary complications, longer fever duration and hospital stay, compared to those receiving conventional doses. Elevated CRP (≥44.45 mg/L), LDH (≥590 IU/L), ferritin (≥411 ng/L), and neutrophil percentage (≥73.75%) were significant predictors for pulse therapy initiation (*p* < 0.05).
Okumura T et al. [33] 2019	Japan	Retrospective cohort study	91 patients aged <15 years with RMPP	Treatment of refractory MPP patients with high-dose corticosteroids (prednisolone equivalents 3.5 ± 1.1 mg/kg/day) could lead to an earlier defervescence (0.8 ± 1.0 vs. 1.5 ± 1.4 days, *p* = 0.01) and shorten hospitalization (8.2 ± 2.4 vs. 10.7 ± 2.7 days, *p* < 0.001) compared with patients treated with low-dose corticosteroids (prednisolone equivalents 1.2 ± 0.3 mg/kg/day). In none of the patients were any corticosteroid-related adverse events observed.
Zhao J et al. [29] 2024	China	Systematic review and meta-analysis	2034 patients aged 3–13 years with MPP	Combined therapy with azithromycin and inhaled budesonide significantly improved overall treatment efficacy across all administration routes (OR = 0.156; *p* < 0.001), without increasing adverse events. It reduced time to fever resolution by 2.0–3.2 days, cough resolution by 2.0–3.8 days, and rales disappearance by 2.0–3.0 days. Inflammatory markers (IL-6, CRP, TNF-α) were significantly decreased (*p* < 0.01), while lung function parameters (FEV1, FVC, PEF) showed significant improvement compared to monotherapy.
Qiu JL et al. [36] 2020	China	Systematic review and meta-analysis	1130 patients aged 4–8 years (with some variations based on individual studies) with RMPP	Glucocorticoids combined with azithromycin can significantly shorten the duration of fever (MD = −2.60; 95%CI −3.11, −2.10; *p* < 0.0001), improve cough symptoms (rale vanishing time: MD = −3.42; 95% CI −4.24, −2.60; *p* < 0.0001; cough recovery time: MD = −3.42; 95% CI −4.05, −2.79; *p* < 0.0001), promote the absorption of pulmonary inflammation at the images (OR = 5.38; 95% CI 1.09, 26.51; *p* = 0.04), shorten hospital stay (MD = −4.63; 95% CI −6.15, −3.17; *p* < 0.0001), reduce the level of inflammatory factors (CRP: MD = −7.17; 95% CI −12.06, −2.28; *p* = 0.004; CD4/CD8: MD = 0.22; 95% CI 0.12, 0.32; *p* < 0.0001). No significant difference in adverse events (*p* = 0.56).
Kim HS et al. [37] 2019	South Korea	Systematic Review and Meta-Analysis of randomized controlled trials	2365 patients aged <18 years with MRMP	The mean duration of fever (WMD = −3.32; 95% CI: −4.16 to −2.48; *p* < 0.00001), length of hospital stay (WMD = −4.03; 95% CI: −4.89 to −3.18; *p* < 0.00001), and CRP levels (WMD = −16.03; 95% CI: −22.56 to −9.50; *p* < 0.00001) were significantly reduced in the glucocorticoid treatment group (methylprednisolone, dexamethasone, or prednisolone combined with macrolides) compared to the conventional treatment group (macrolide monotherapy). However, all outcome measures showed high heterogeneity, and sensitivity analyses did not confirm a significant difference.
Sun LL et al. [41] 2020	China	Meta-analysis	1049 patients aged 9 months–12 years with SMPP	High-dose methylprednisolone significantly improved clinical effectiveness (RR = 1.30; 95% CI: 1.23–1.38; *p* < 0.05) compared to low-dose therapy. It also resulted in shorter time to temperature recovery (MD = −2.71 days; 95% CI: −3.59 to −1.83; *p* < 0.00001), hospital stay (MD = −3.70 days; 95% CI: −6.17 to −1.23; *p* < 0.003), pulmonary rales resolution (MD = −2.50 days; 95% CI: −3.38 to −1.63; *p* < 0.00001), cough disappearance (MD = −2.39 days; 95% CI: −2.99 to −1.79; *p* < 0.00001), and pulmonary shadow absorption (MD = −5.34 days; 95% CI: −6.89 to −3.78; *p* < 0.00001). There was no significant difference in the incidence of adverse events between the two groups (RR = 0.85; 95% CI: 0.53–1.36; *p* > 0.05).

**Abbreviations:** CI, confidence interval; CRP, C-reactive protein; CT, computed tomography; ESR, erythrocyte sedimentation rate; FeNO, fractional exhaled nitric oxide; FEV1, forced expiratory volume in 1 s; FVC, forced vital capacity; HRCT, high-resolution computed tomography; IL-6, interleukin-6; IV, intravenous; IVIG, intravenous immunoglobulin; LDH, lactate dehydrogenase; MD, mean difference; MRMP, macrolide-resistant *Mycoplasma pneumoniae*; MPP, *Mycoplasma pneumoniae* pneumonia; PEF, peak expiratory flow; RMPP, refractory *Mycoplasma pneumoniae* pneumonia; RR, risk ratio; SMPP, severe *Mycoplasma pneumoniae* pneumonia; TNF-α, tumor necrosis factor alpha; WMD, weighted mean difference.

**Table 2:** Steroid dose equivalence and clinical application across studies.

Study	Clinical Syndrome	Type of Corticosteroid Molecule Used in the Study	Hydrocortisone Dose Equivalence
You SY et al. [12] 2013	Refractory *Mycoplasma pneumoniae* pneumonia	Intravenous (IV) methylprednisolone pulse therapy at 30 mg/kg/day for 3 days.	150 mg/kg/day
Yang EA et al. [13] 2019	*Mycoplasma pneumoniae* pneumonia	Oral prednisolone 1 mg/kg/day (for mild cases) IV methylprednisolone 1–2 mg/kg/day (for mild cases) IV methylprednisolone 5–10 mg/kg/day (for severe cases)	4 mg/kg/day 5–10 mg/kg/day 25–50 mg/kg/day
Zhang H et al. [20] 2023	*Mycoplasma pneumoniae* pneumonia	Budesonide inhalation 1 mg	Non-feasibility equivalence
Liu J et al. [25] 2023	*Mycoplasma pneumoniae* pneumonia	Methylprednisolone 5–15 mg/kg/day Methylprednisolone 5–30 mg/kg/day	25–75 mg/kg/day 25–150 mg/kg/day
Zhao Q et al. [26] 2022	*Mycoplasma pneumoniae* pneumonia	Methylprednisolone first dose 2 mg/kg/day, after 3–5 days 1 mg/kg/day for 3 days	10 mg/kg/day 15–25 mg/kg/day
Huang L. et al. [27] 2014	*Mycoplasma pneumoniae* pneumonia	Methylprednisolone 2 mg/kg/day within 24 h, followed by a 1-week prednisolone taper	10 mg/kg/day
Han HY et al. [28] 2021	*Mycoplasma pneumoniae* pneumonia	Oral prednisolone 1 mg/kg/day IV methylprednisolone 1–2 mg/kg/day IV methylprednisolone 5–10 mg/kg/day	4 mg/kg/day 5–10 mg/kg/day 25–50 mg/kg/day
Zhao J et al. [29] 2024	*Mycoplasma pneumoniae* pneumonia	Budesonide	Non-feasibility equivalence
Zhou H et al. [30] 2022	Refractory *Mycoplasma pneumoniae* pneumonia	IV methylprednisolone 2 mg/kg/day for 5 days, then 1 mg/kg/day for 2 days	10 mg/kg/day 5 mg/kg/day
Okumura T et al. [33] 2019	Refractory *Mycoplasma pneumoniae* pneumonia	IV methylprednisolone 2 mg/kg/day up to a maximum dose of 60 mg/d for 3 days, followed by tapering over 12 days (low-dose). IV methylprednisolone increased to 4 mg/kg/day on day 2 for 3 days, followed by tapering over 12 days. IV methylprednisolone 10 mg/kg/day up to a maximum dose of 300 mg/d for 3 days, followed by tapering over 12 days (high dose).	10 mg/kg/day up to a maximum dose of 300 mg/day 20 mg/kg/day 50 mg/kg/day up to a maximum dose of 1500 mg/day
Shan LS et al. [34] 2017	Refractory *Mycoplasma pneumoniae* pneumonia	IV methylprednisolone 2 mg/kg/day for 3 days	10 mg/kg/day
Luo Z et al. [35] 2014	Refractory *Mycoplasma pneumoniae* pneumonia	Oral prednisolone 2 mg/kg/day in two divided doses	8 mg/kg/day
Qiu JL et al. [36] 2020	Refractory *Mycoplasma pneumoniae* pneumonia	Not specified as systematic review and meta-analysis	Not specified as systematic review and meta-analysis
Kim HS et al. [37] 2019	Macrolide- refractory *Mycoplasma pneumoniae*	Not specified as systematic review and meta-analysis	Not specified as systematic review and meta-analysis
Zhu R et al. [38] 2022	Severe *Mycoplasma pneumoniae* pneumonia	IV methylprednisolone 1–2 mg/kg/day for 3–5 days.	5–10 mg/kg/day
Fang C et al. [39] 2022	Severe *Mycoplasma pneumoniae* pneumonia	IV methylprednisolone 10 mg/kg/day for 5 days	50 mg/kg/day
Sun LL et al. [41] 2020	Severe *Mycoplasma pneumoniae* pneumonia	Not specified as systematic review and meta-analysis	Not specified as systematic review and meta-analysis

**Abbreviations:** IV, intravenous.

## Data Availability

No new data were created or analyzed in this study. Data sharing is not applicable to this article.

## Abbreviations

The following abbreviations are used in this manuscript:

BID	Bis in die (twice a day)
CAP	Community-Acquired Pneumonia
CRP	C-Reactive Protein
CSF	Cerebrospinal Fluid
CT	Computed Tomography
DNA	Deoxyribonucleic Acid
FEV1	Forced Expiratory Volume in 1 Second
FVC	Forced Vital Capacity
ICU	Intensive Care Unit
IL	Interleukin
IU	International Units
IVIG	Intravenous Immunoglobulin
LDH	Lactate Dehydrogenase
MIRM	*Mycoplasma pneumoniae*-Induced Rash and Mucositis
MP	*Mycoplasma pneumoniae*
MPP	*Mycoplasma pneumoniae* Pneumonia
MRMP	Macrolide-Resistant *Mycoplasma pneumoniae*
PCR	Polymerase Chain Reaction
PCT	Procalcitonin
PEF	Peak Expiratory Flow
PEF25	Peak Expiratory Flow at 25%
RIME	Reactive Infectious Mucocutaneous Eruption
RMPP	Refractory *Mycoplasma pneumoniae* Pneumonia
SMPP	Severe *Mycoplasma pneumoniae* Pneumonia
TNF	Tumor Necrosis Factor
